# Fitness for Military Service

**Published:** 1918-04-06

**Authors:** 


### FITNESS FOR MILITARY SERVICE.

In the severe inquisition to which the man-power of the nation is at present being subjected the doctors charged with the duty of examining men with a view to military service have a very difficult and responsible task. Moreover, the duty is sometimes a distinctly painful one. It is no light and easy decision to order a young man whose whole inclination is towards peaceful pursuits to take up the strain and risks of active service in the field. Perhaps the most acute position is that of the doctors in charge of military hospitals, for these repeatedly have to decide the question here at issue in reference to men who have already been in the field and who have been sent home in consequence of wounds or illness. These men have actually suffered the dangers of the war, and obviously, did circumstances permit it, one would be glad to release them from further exposure. But these natural leanings must be held in check. The medical duty is to pronounce these men either as fit or unfit, and the consequences of the decision rest with the military authorities as these are directed by the law of the land. Now there are, of course, cases not a few in which unfitness is obvious. Leaving on one side loss of limbs and other serious mechanical injuries, there are among the military hospital population cases of conspicuous cardiac disease, valvular or otherwise, of pulmonary tuberculosis, of exophthalmic goitre, of epilepsy, and so on. On these and kindred conditions there is no room for any difference of opinion. The facts are insistent, overt, conspicuous, and admit of but one interpretation. For military purposes such patients are useless, and they are naturally therefore certified to be discharged from the Army. What will be their future may be an exercise in medical prognosis, but with this the military organisation has no concern.

The difficulty for the medical officer is therefore not with the cases just mentioned but with cases in which, while physical evidences of disease are absent, the patient declares himself to be unable for the proposed task. He has been ill, let us say, with trench fever or with pneumonia or with some more or less similar condition. But the febrile disturbance has passed away and the man is convalescent. Yet he complains of such symptoms as pains or weakness, or palpitation or breathlessness; and exertion is advanced as the exciting or aggravating cause of these conditions. Hence the doctor is faced with the difficult problem of deciding the immediate future of the man and with little or no evidence to guide him save the man's own statements; and clearly, in such circumstances, these statements cannot be treated as conclusive evidence. What must happen under such conditions is that individual medical officers charged with the duty of judgment will come to different conclusions, for there is no certain and common standard to direct them. They are called on to estimate the man's abilities for certain tasks, and they have only very vague and general considerations to guide them. The prognosis expected is, it is true, very limited as regards time, but it is very heavily burdened with immediate responsibilities. An incorrect judgment necessarily means either that the country is deprived of the services of a competent man or the man is set to do tasks which break down his health. The debate, be it plainly understood, is not concerned with obvious and gross disease. It relates to men who, as tested by an ordinary clinical examination, would be pronounced to be sound, and the doctor has to say whether or not the man is fit for one or other grade of Army service.

In reference to this order of cases a large and valuable series of observations has been accumulated by Dr. Thomas Lewis from his experience at the Military Heart Hospital at Colchester. Dr. Lewis has published his results in the *Lancet* of February 2. The system pursued, whilst it increases the supply of man-power for military purposes, tends to protect the individual who is really unfit but who, being free from signs of organic disease, is likely to be regarded as a more or less suitable man to be returned to the Front. The figures in this respect are impressive, for they show that out of a total of 558 men tested in the manner Dr. Lewis describes no fewer than 286 were discharged as permanently unfit for service.

